# Comparing human text classification performance and explainability with large language and machine learning models using eye-tracking

**DOI:** 10.1038/s41598-024-65080-7

**Published:** 2024-06-21

**Authors:** Jeevithashree Divya Venkatesh, Aparajita Jaiswal, Gaurav Nanda

**Affiliations:** 1 School of Engineering Technology, Purdue University, West Lafayette, IN 47907 USA; 2 Center for Intercultural Learning, Mentorship, Assessment and Research (CILMAR), Purdue University, West Lafayette, IN 47907 USA

**Keywords:** Human-AI alignment, Large language models, Explainable AI, Eye tracking, Cognitive engineering, Human–computer interaction, Human behaviour, Software, Classification and taxonomy

## Abstract

To understand the alignment between reasonings of humans and artificial intelligence (AI) models, this empirical study compared the human text classification performance and explainability with a traditional machine learning (ML) model and large language model (LLM). A domain-specific noisy textual dataset of 204 injury narratives had to be classified into 6 cause-of-injury codes. The narratives varied in terms of complexity and ease of categorization based on the distinctive nature of cause-of-injury code. The user study involved 51 participants whose eye-tracking data was recorded while they performed the text classification task. While the ML model was trained on 120,000 pre-labelled injury narratives, LLM and humans did not receive any specialized training. The explainability of different approaches was compared based on the top words they used for making classification decision. These words were identified using eye-tracking for humans, explainable AI approach LIME for ML model, and prompts for LLM. The classification performance of ML model was observed to be relatively better than zero-shot LLM and non-expert humans, overall, and particularly for narratives with high complexity and difficult categorization. The top-3 predictive words used by ML and LLM for classification agreed with humans to a greater extent as compared to later predictive words.

## Introduction

Text classification has a wide variety of applications including spam filtering of emails, organization of documents and articles, and analyzing electronic health reports. Most of the automated text classification happens through supervised machine learning (ML) models. These models are trained on human-annotated training data to learn how the features (words or phrases) in the text are predictive of certain classification categories and use that learning to predict the category for a new text record. The accuracy of ML models is highly dependent on the accuracy and consistency of the human labelling or annotation, which is not always perfect due to variation in human performance, expertise in classification rules, and interpretation of the text. Another reason for limited prediction accuracy in traditional ML models is the lack of usage of semantic and syntactic information of the text. Recent advancements in natural language processing (NLP), such as word embeddings, transformers, and large language models (LLMs) have better capabilities in terms of understanding and utilization of syntactic and contextual semantic information of the text^1–3^, which is likely to improve the classification performance.

## Explainability in text classification

With the increasing usage of ML models in diverse domains, explainability has emerged as an important aspect for understanding the rationale behind classification decisions made by humans or ML models. In the context of ML models, simpler ML models, such as Naïve Bayes and decision trees, are often not highly accurate but can be easily understood by end-users due to their relatively simple structure or mathematical formulation. On the other hand, more sophisticated models, such as neural networks, gradient-boosted decision trees, and ensemble models (e.g., random forest classifiers), typically have better predictive power but are difficult to understand because of their complex formulations or the proprietary nature of their learning algorithms. To render these models more interpretable and explainable to their end-users, several explainable AI tools have been developed recently^4^ that can provide feature-based or data-based explanations of the outcomes of ML models and process. Feature-based approaches, such as LIME^5^ and SHAP^6^ identify the most important features responsible for the model’s outcomes either for the entire dataset (global explanations) or for a particular data instance (local explanations). For instance, SHAP measures the marginal contribution of features to the difference between a particular prediction and the average model prediction^6^. LIME, on the other hand, provides local explanations for a model’s prediction on a data instance; for a given instance, it probes the black-box model with a new data instance obtained by perturbing the given instance, obtains the model predictions, and repeats this process to learn a surrogate, interpretable model (e.g., linear regression or decision tree) in the neighborhood of that instance^5^.

While the aforementioned explainability approaches have become popular because of their applicability to multi-modal, multi-class classification settings, explainability of text classification by humans and LLMs has not been well-studied. Extending these XAI for LLMs is challenging due to the large number of model parameters (175 billion for GPT-3 model)^7^. Additionally, while manual text classification is the primary method of annotating cause-of-injury codes, information about the reasoning behind the category selected for each case by the human annotator is typically not recorded, as it is a time- and resource-consuming process. As such, the explainability aspects of manual text classification in the context of injury surveillance have not been well-studied.

## Study objectives

In this study, we aim to fill these gaps in the literature by studying and comparing the performance and explainability aspects of text classification performed by humans, traditional ML model, and LLMs. For human explainability analysis, we performed eye-tracking user study in which the participants performed text classification and the words on which they focused were identified. For LLM explainability analysis, we used prompt-based questions asking ChatGPT-3.5^7^ to list the top words in the narrative used for decision making. For ML model, we used the explainable AI approach LIME^5,6^ to identify the top words used by the model during classification. The text classification task used in this study was assigning external cause-of-injury codes^8–10^ to accident narratives collected at hospital emergency rooms. The nature of text was not very generic in nature (e.g., customer reviews of products or services) but somewhat domain-specific with narratives containing injury related medical terms. The noisy nature of narratives with misspellings and improper sentence structures, and lack of clarity about the underlying cause of injury made the text classification task moderately challenging.

## Related work on eye tracking

Previous studies have used eye tracking for analyzing human text comprehension^11,12^ as eye tracking while reading is considered as one of the most informative physiological data channels^14^. While reading, humans move their gaze position from word to word in a line of text, resulting in two main types of movement patterns: rapid eye movements known as saccades, and points of gaze movement pauses called fixations. During fixations, the human mind actively processes the meaning of the text^15^ providing information about how long a person spent looking at the text, which words were skipped^16^, and so on. Thus, eye tracking data recorded during text reading provides objective insights about reading patterns and duration of visual attention assigned to specific parts of the text at the level of phrase, word, or character^13,17^.

In addition to reading and language comprehension, eye-tracking has also been used in understanding how humans perform other linguistic tasks such as annotation^18,19^. Previous studies^20,21^ have analyzed eye tracking data such as number of fixations, search time, and fixation duration recorded during manual annotation of named-entities in texts to a) estimate the difficulty level based on cognitive load assessed using gaze data eye-tracking data, and, b) identify prominent features used by humans for named-entity recognition. Some studies^22–24^ have tried to develop cognitive models using eye tracking data for estimating reading and manual sentiment annotation complexity, and reported that fixation duration can be predictive of sentiment annotation complexity. There have also been some recent studies^25,26^ that have developed neural networks integrating the text features and eye tracking data for various natural language processing (NLP) tasks such as determining sentiment polarity and identifying sarcasm.

## Injury narrative classification: overview and challenges

In this study, we have focused on the text classification task of assigning six cause-of-injury codes (CUT, FALL, STRUCK, BURN, MOTORVEHICLE, and OTHER) to accident narratives. It is similar to injury coding, in which the unstructured incident narratives collected at hospitals and injury reports are transformed into structured form by assigning different types of injury codes, such as cause-of-injury, product-involved, and nature-of-injury. It is an important step in the injury surveillance and prevention efforts carried out by public health agencies as it enables statistical analysis of structured data to identify trends and patterns^27^. Injury coding is similar to other multi-label text classification tasks, but following aspects make it challenging, (a) the narratives are short and noisy text snippets with misspellings and non-grammatical sentences, and (b) there are a large number of injury codes with only few dominant ones leading to imbalanced data distribution^8,28^.

Traditionally, injury coding has been done manually where the human coders assign injury codes based on the narratives. Manual coding is time and resource consuming and also involves quality challenges, such as (a) subjective interpretation of text by different individuals leading to inconsistent coding, (b) incorrect assignment of low-frequency or rare injury codes typically by less-experienced coders^8,27^. In recent years, ML models trained on manually coded historical injury records have been used to predict the injury codes^8,9,28^. While these ML models have showed decent prediction accuracy overall and for high-frequency injury codes, their accuracy for low-frequency injury codes has been limited, primarily due to inconsistencies in training data and the noisy nature of narratives^8,27,28^.

## Study design overview

The dataset of injury narratives used in this study was generously provided by the Queensland Injury Surveillance Unit (QISU)^29^, which contained the accident narratives collected from hospital emergency rooms and the associated cause-of-injury codes assigned by professional coders. For human text classification analysis, we conducted an eye tracking user study involving 51 participants who had to perform a text classification task of assigning cause-of-injury codes to accident narratives while their eye-tracking data was recorded. Each participant had to read 12 accident narrative prompts on a computer screen and select the most appropriate external cause-of-injury code for each prompt from a list of six possible cause-of-injury codes: CUT, FALL, STRUCK, BURN, MOTORVEHICLE, and OTHER. The set of 12 prompts (later referred as prompt-set) consisted of 2 unique accident narratives belonging to each cause-of-injury code, but the participants were unaware about the equal number of prompts for each injury code.

The total user study dataset consisted of 3 repetitions of 17 unique prompt-sets that were used for 51 (= 17*3) participants. Thus, for each prompt-set, data was collected from 3 participants with the intention to account for human variability. The overall dataset included 34 (= 17*2) unique narrative cases for each of the 6 cause-of-injury codes, i.e., a total of 204 (= 34*6) unique narrative cases. These narrative cases were derived from the QISU database from the year 2018 and the cause-of-injury codes were assigned by professional QISU coders. During the study, each participant’s ocular parameters- fixation count (FC) and fixation duration (FD), were recorded with the resolution-level of individual word in the narrative while they performed the text classification task. The eye-tracking data was analyzed to identify which words users focused on (i.e., words with highest fixation count and fixation duration) while reading and comprehending the narrative and making the selection of most applicable cause-of-injury code. The top words were selected based on the highest FC and FD values.

For ML, we used the Logistic Regression (LR)^30^ model trained on a non-overlapping training dataset of 120,000 injury cases to predict the cause-of-injury code on the above mentioned 204 cases test dataset. We used the explainability approach of LIME based ELI5^5^ to identify the top-5 words in the narrative that the LR model used. For LLM, we used ChatGPT-3.5 to predict the cause-of-injury code in a zero-shot learning manner (i.e., without any training) on the same 204 case test dataset. For explainability analysis of LLMs, we used prompts to identify the top-10 words used by the model and used perturbation-based attribution approach^31,32^ to validate the predictive capabilities of the top words outputted by the LLM. The intention behind using zero-shot learning approach for LLM was to replicate the performance of a non-expert human coder who has a reasonable understanding of the language but does not have specialized training on injury classification, which requires some domain-specific knowledge.

## Results

**Figure 1:**
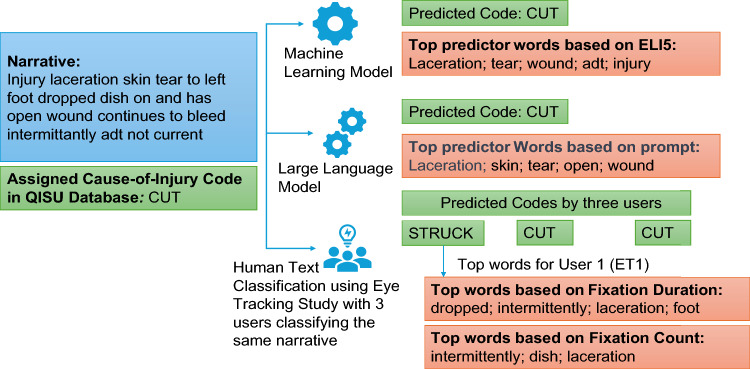
Overview of study showing classification and explainability results for a sample narrative.

For the human text classification eye tracking user study, the participant-selected cause-of-injury codes for each narrative in 204-case dataset was recorded. As explained in the study design, each case in the dataset was analyzed by three participants (17 unique sets * 12 case per set* 3 batches = 204). ET1, ET2 and ET3 are referred to as the eye tracking data recorded for 3 batches of cases across 51 participants. For the ML and LLM models, the cause-of-injury codes predicted by the LR and ChatGPT-3.5 models for each narrative in the 204-case dataset were recorded. The output for a sample case used in the study is shown in Fig. 1 below, depicting the injury narrative, the originally assigned cause-of-injury code in QISU database, the predicted cause-of-injury code and top predictor words for the ML model, the LLM, the predicted cause-of-injury code by the three human study participants, and the top words for one human participant based on fixation count and fixation duration obtained from eye-tracking recording.

## Comparing text classification performance between Humans, ChatGPT, and ML

The text classification performances of the three approaches (human, ML, and ChatGPT) were evaluated on the set of 204 injury cases by comparing the cause-of-injury codes predicted by eye-tracking study participants, ML model, and ChatGPT with the original codes assigned by the QISU professional coders. Recall was used as the primary measure of performance for each of the six cause-of-injury codes as well as overall performance on the whole dataset, as described in Eq. (1):

1 $$Recall =\frac{True\,positive\,predicitons}{Total\,number\,of\,cases}$$

**Figure 2:**
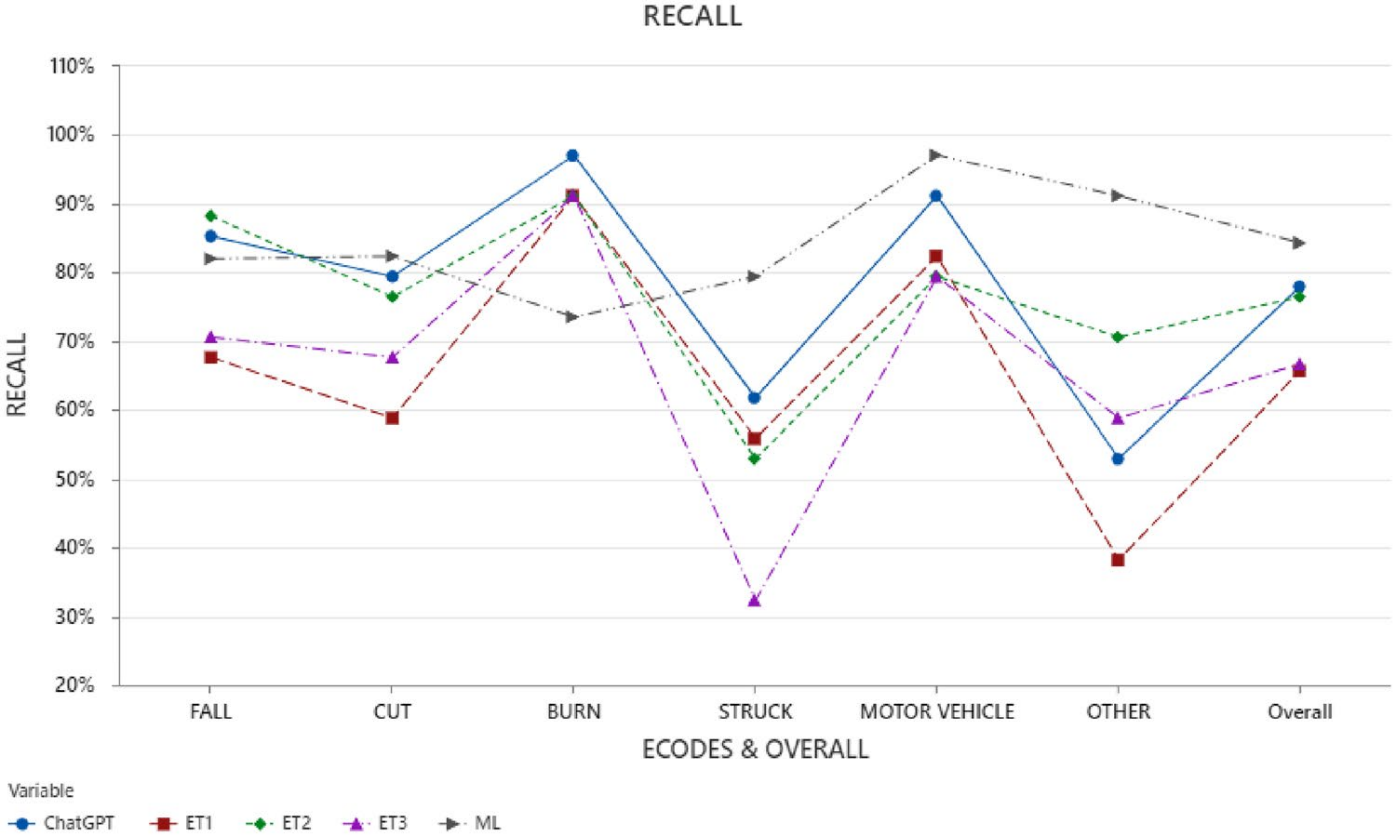
Recall for 6 cause-of-injury codes.

For Recall calculations, a case was counted as True Positive when the predicted cause-of-injury code agreed with the originally assigned QISU code, and the total number of cases (in the denominator of Eq. 1) for individual cause-of-injury code was 34 and for the overall Recall it was 204 (size of the dataset). Due to space considerations, other commonly used performance measures such as Precision or F1-scores are not included in the paper. Figure 2 presents the Recall for each injury code and the overall dataset for Human study (three sets- ET1, ET2, and ET3), ChatGPT, and ML.

As shown in Fig. 2, among the three text classification approaches, the ML model reported the highest overall Recall (84.3%) as compared to Human and ChatGPT. One of the possible underlying reasons for this may be that the ML model was trained exclusively on a large dataset of injury narratives. For baselining purposes, we also compared the prediction performance of LR with other traditional ML models, and observed their overall Recall as following: Random Forest: 83.3%; Support Vector Machine: 85.8%; and Multinomial Naïve Bayes: 78.9%.

On the other hand, while Human study participants and ChatGPT had capabilities of general understanding of the narrative text and cause-of-injury category definitions, they did not have specific training or experience on injury narrative classification task to understand the nuances. The text classification study participants did not have any significant prior knowledge on injury data analysis and the LLM ChatGPT although being trained on several textual databases from different domains, has not been exclusively trained on injury-code related datasets. Figure 2 also shows the variations in Humans’ text classification performance across the 3 sets ET1, ET2, and ET3 among the 51 study participants. It is to be noted that there were multiple participants involved in each of the user study batches- ET1, ET2, and ET3, therefore, a comparative analysis between the batches was not performed.

## Variation in performance based on the distinct nature of the code

Among the different cause-of-injury codes, the classification performance varied based on the uniqueness of the code, i.e., how distinct were the codes relative to other codes so that they are less likely to be confused with another code. This was particularly important for Human and ChatGPT approaches where specialized training on injury coding was not provided. As the ML model was trained on thousands of cases of each injury code, it was expected to learn the classification rules in a more detailed manner. The cause-of-injury codes BURN and MOTORVEHICLE were relatively unique in nature, codes FALL, STRUCK and CUT were moderately unique, and the code OTHER was not very unique in nature. The injury code descriptions are provided in Table 1 in the Methods section. The distinct nature of codes BURN and MOTORVEHICLE meant that the narratives typically included a unique set of words, for example, in describing the type of injury (e.g., *burned* or *scalding* for BURN) or the product involved (e.g., *car* or *pedestrian* for MOTORVEHICLE), that did not overlap with narratives of other injury codes. On the other hand, the injury codes that were not very unique in nature contained overlapping elements with other injury codes or did not have clear definitive classification rules. For example, the codes CUT and STRUCK had some overlapping elements such as interaction between a tool and a person, and the code OTHER had a relatively fuzzy definition that the narrative does not belong to any other cause-of-injury codes.

**Table 1:** Description for injury event cause groups.

Injury event cause group	Description
Fall	Fall on some level or height
Struck	Struck by or collision with person or object
Cut	Cutting or piercing by object
Burn	Burn due to hot object, fluid, or gas including hot drink, food, water, other fluid, steam, gas, and other types of contact burns
Motor Vehicle	Any type of motor vehicle accident involving driver or passenger
Other	Any other cause of injury not including Fall, Struck, cut, Burn or Motor Vehicle

As shown in Fig. 2, codes BURN and MOTORVEHICLE, which had relatively unique definitions, reported higher Recall for Humans and ChatGPT as compared to other injury codes. It is also to be noted that there was lesser variation in the performance of the three Human sets ET1, ET2, and ET3 for BURN and MOTORVEHICLE as compared to other injury codes. This may be indicative that the text classification process for these codes was less confusing for non-experts due to their unique nature. For BURN, ChatGPT (97%) and Humans (~ 91% for all three sets) reported their highest Recall. It is also interesting to note that while ML reported its highest Recall for MOTORVEHICLE (97%), it reported its lowest Recall for BURN (74%).

Humans and ChatGPT reported relatively low Recall for cause-of-injury codes OTHER, CUT, and STRUCK, while the ML model performed relatively better for these categories. It is also to be noted that the variation in human sets ET1, ET2, and ET3 is relatively large for these categories. For cause-of-injury code OTHER, ML reported highest Recall (79%), followed by Humans (ET1-38%, ET2-71%, ET3-59%) and ChatGPT (53%). For STRUCK, the Recall for ML (79%) was significantly high as compared to Humans (ET2- 53%, ET3- 32%) and ChatGPT (62%). All three approaches- Humans, ChatGPT and ML, reported their lowest individual Recall for STRUCK. For CUT, the Recall was close for ML (82%) and ChatGPT (79%) followed by Humans (ET1-59%, ET2-76%, ET3-68%). It is also to be noted that the ML model reported better Recall than Human and ChatGPT for all cause-of-injury codes, except FALL and BURN. For FALL, the ML Recall was marginally less than ChatGPT and ET3. For FALL cause-of-injury code, Humans reported highest Recall (ET2-88%) as well as lowest Recall (ET3-59%).

## Variation in prediction performance based on complexity of narratives

**Figure 3:**
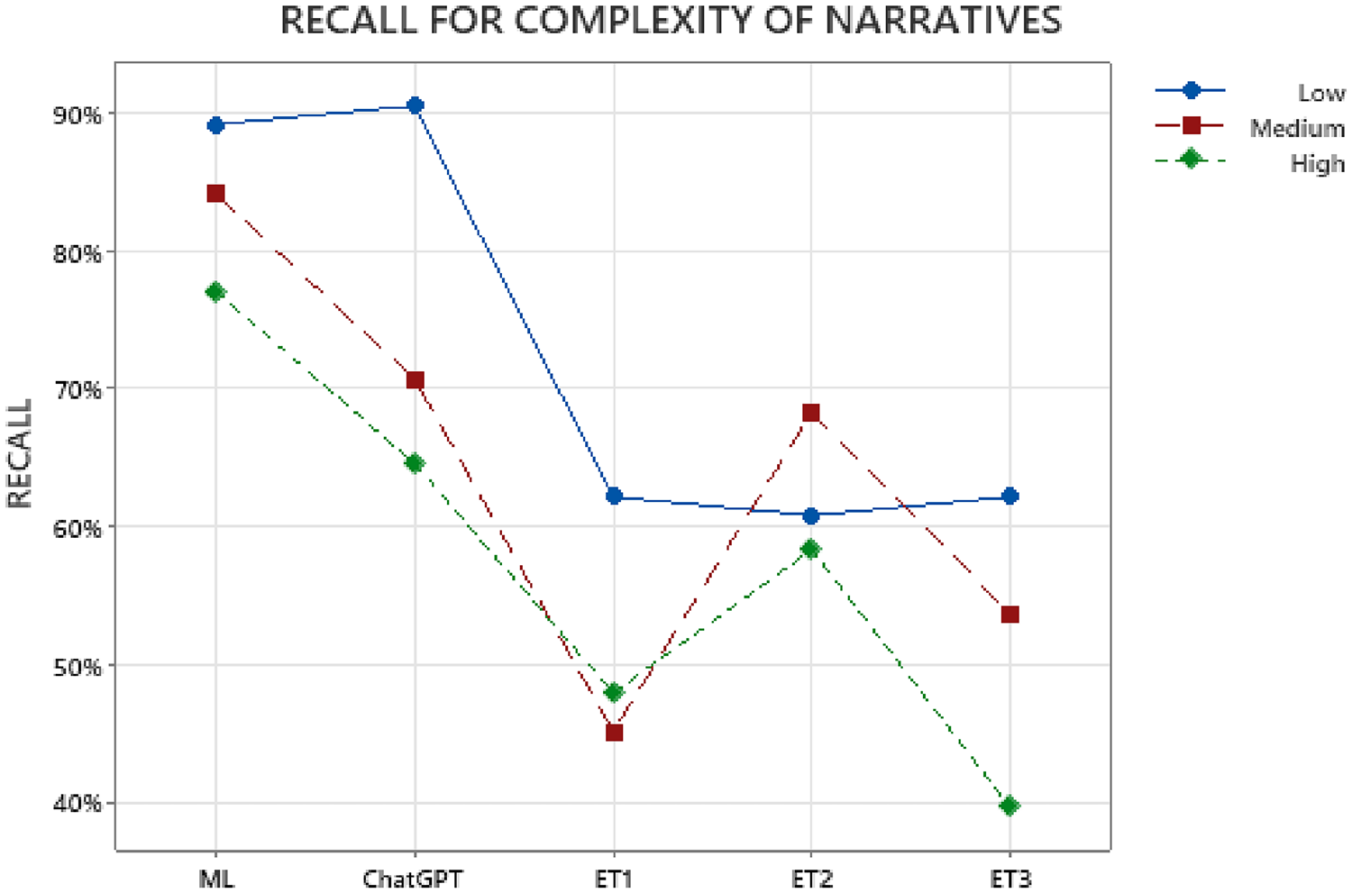
Variation in Text Classification Recall of Humans (ET1, ET2, ET3), ML and ChatGPT based on Complexity of Narratives.

Each of the narratives in the set of 204 prompts used for classification task were internally classified into 3 levels of complexities-Low, Medium, and High, based on two factors: (a) the level of difficulty in comprehending the narrative text and (b) the lack of clarity or obviousness in selecting the most appropriate cause-of-injury code either due to multiple possible codes or lack of information in narrative. The eye-tracking study participants were not aware of these different narrative complexity levels during their text classification task. Overall, out of the 204 narratives, there were 74 narratives categorized as “Low”, 82 narratives categorized as “Medium” and 48 narratives categorized as “High” complexity. We analyzed the variation in Recall values based on narrative complexity for the predictions made by Humans (ET1, ET2, ET3), ChatGPT, and ML, presented in Fig. 3.

As shown in Fig. 3, the highest Recall values were observed for narratives with low complexity for ML (89%), ChatGPT (91%), and Humans- ET1 (62%) and ET3(62%). However, ET2 reported highest Recall (68%) for narratives with “Medium” complexity. Overall, considering the variations in human performance between groups ET1, ET2, and ET3 shown in Fig. 3, we can observe that, a) for low complexity narratives, ML and ChatGPT performed considerably better than humans, b) for medium and high complexity narratives, ML performed considerably better than ChatGPT and humans, and ChatGPT performed marginally better than humans.

## Explainability: comparing top words between humans, ChatGPT, and ML

We also studied the reasoning behind the text classification choices made by humans, traditional ML model, and LLM (ChatGPT) by studying the words in narrative text that were used by them for classification decision making. For human text classification (ET1, ET2, ET3), word-level eye tracking parameters- FC and FD were recorded and analyzed for each participant to identify the top-10 words that participants focused on while comprehending the narrative and selecting the cause-of-injury code. As a further validation step for eye-tracking data, we collected think-aloud recordings for each participant, in which each participant was asked to verbally explain their reasoning for choosing that injury code for each record, and their voice was recorded and transcribed. The alignment of reasoning between the top eye tracking words and think-aloud transcription was qualitatively analyzed. We examined the overlap between the words mentioned in think-aloud by participants that described their reasoning for classification and the top words identified by eye tracking based on highest fixation count and fixation duration values for each case. Cases with overlap of three or more words were categorized as ‘high’ alignment, those with two words were marked as ‘medium’ alignment, and fewer than two words were categorized as ‘low’ alignment. Results indicated that 52% cases were of high alignment, 19% cases had medium alignment, and 29% cases observed low alignment. With 71% cases observing high/medium alignment, it demonstrates that the eye-tracking based technique was able to capture human reasoning reasoning well in terms of top predictive words.

For the ML model, the top-5 words in the narrative used by the LR model for making the prediction were determined using LIME based ELI5 explainability analysis. The top-5 ML words are referred to later in the paper as ML1-(top 1st word), ML2 (2nd word), ML3 (3rd word), ML4 (4th word), and ML5 (top 5th word). For the LLM, the top-10 words used by the ChatGPT model were obtained through prompts asking to list the top words used by the model for text classification decision making in decreasing order of importance. We performed additional prompt-based perturbation analysis to validate the predictive strength of top words outputted by LLM. We studied the impact of omitting the top-1 and top-3 words from the narrative on the prediction performance of LLM. When the top predictive word (top-1) was omitted from the narrative text, the overall Recall of ChatGPT dropped from 77 to 53% and when the top-3 words were removed, it dropped further down to 34%. This indicated that the top 3 words provided by the LLM had considerable predictive weightage.

**Figure 4:**
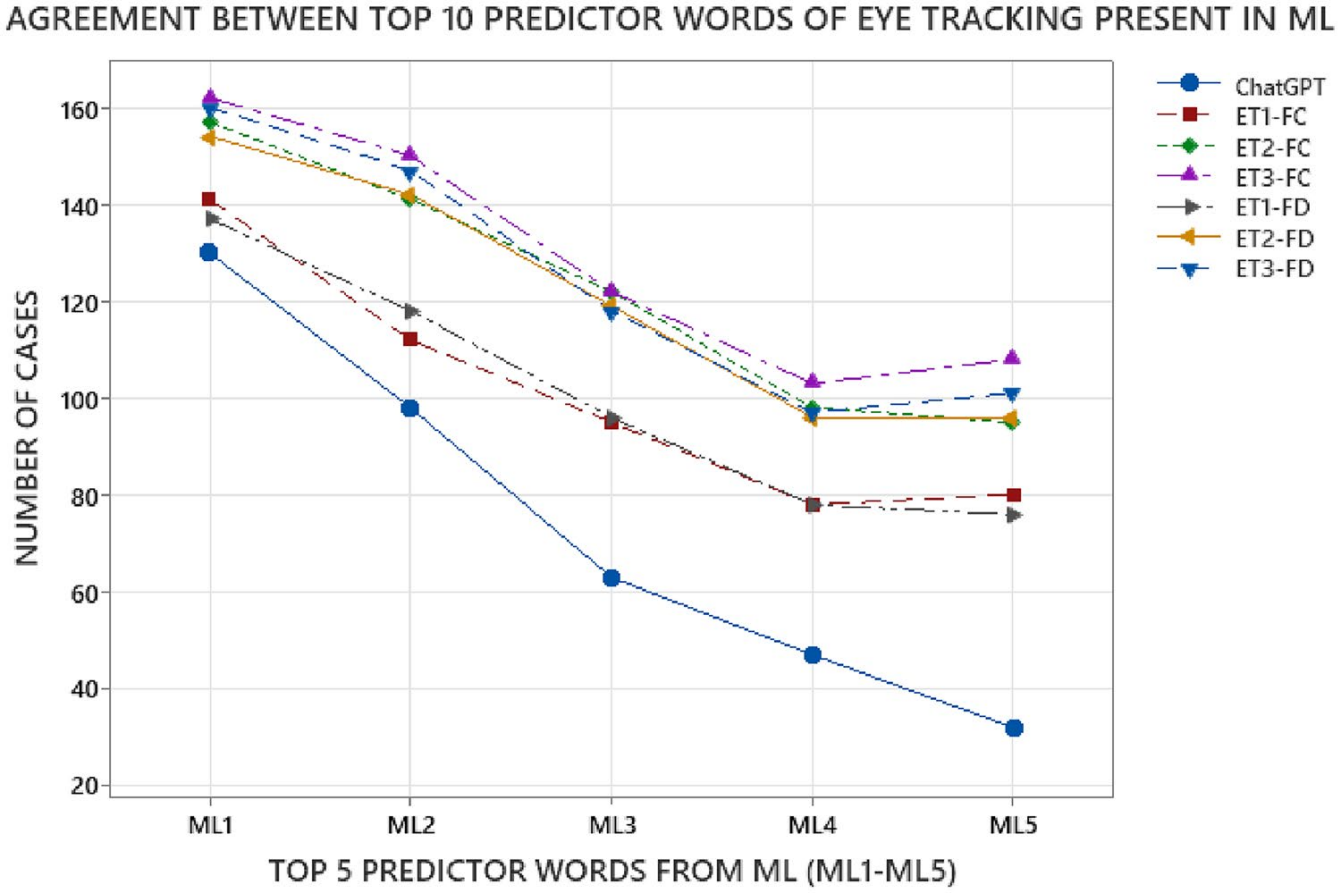
Agreement of top 10 predictor words of eye tracking (ET) present in machine learning (ML).

Next, we compared the level of agreement between the top predictor words of ML model with humans and ChatGPT. The level of agreement between the top words of humans and ML model was calculated by comparing the overlap between the top-5 words of ML model with top-10 predictor words of humans for each of the 206 cases in the dataset. Similarly, the level of agreement between the top words of ML and ChatGPT was calculated by comparing the overlap between top-5 words of ML and the top-10 words used by ChatGPT for each case in the dataset. Figure 4 shows the agreement level for each of the top-5 ML words (ML1-ML5) with humans (ET-1, ET-2, ET-3 based on fixation count (FC) and fixation duration (FD)) and ChatGPT. The Y-axis represents the number of cases out of total 206 dataset where the top-nth word of ML model was present in the top-10 word lists of ChatGPT and humans (ET-FCs and ET-FDs). The X-axis shows the top ML words (ML1-ML5).

As shown in Fig. 4, the topmost ML word– ML1 overlapped with ET and ChatGPT top-10 words for maximum number of cases in the dataset, followed by next top words ML2, ML3, ML4, and ML5. This behavior was somewhat expected as the top predictor words provided by ELI5 in decreasing order of importance. Similarly, the list of top-10 predictor words ChatGPT were also organized in decreasing order of importance as it was instructed to do so in the prompts. One of the interesting trends to be observed in Fig. 4 is that there was a relatively substantial decline in the number of cases with matching top-words between ML and other approaches after ML2, as compared to the decline from ML1 to ML2. This indicates that the top-2 ML predictor words were considerably more predictive as compared to ML3, ML4, and ML5.

Another noticeable trend in Fig. 4 was that the decline in agreement between the top words of ML and ChatGPT going from ML1 to ML5 was considerably steeper as compared to the decline in agreement of top words of ML and humans (ET-FCs and ET-FDs). This relatively higher overlap between the top words of ML and humans indicates that there was better alignment in the reasoning of classification choices between ML and humans as compared to ML and ChatGPT. We can also observe in Fig. 4 that there was a slightly higher agreement level with humans for ML5 as compared to ML 4, which might be indicative that there may not be as significant difference in the importance scores of ML4 and ML5 as compared to the topmost words ML1 and ML2.

**Figure 5:**
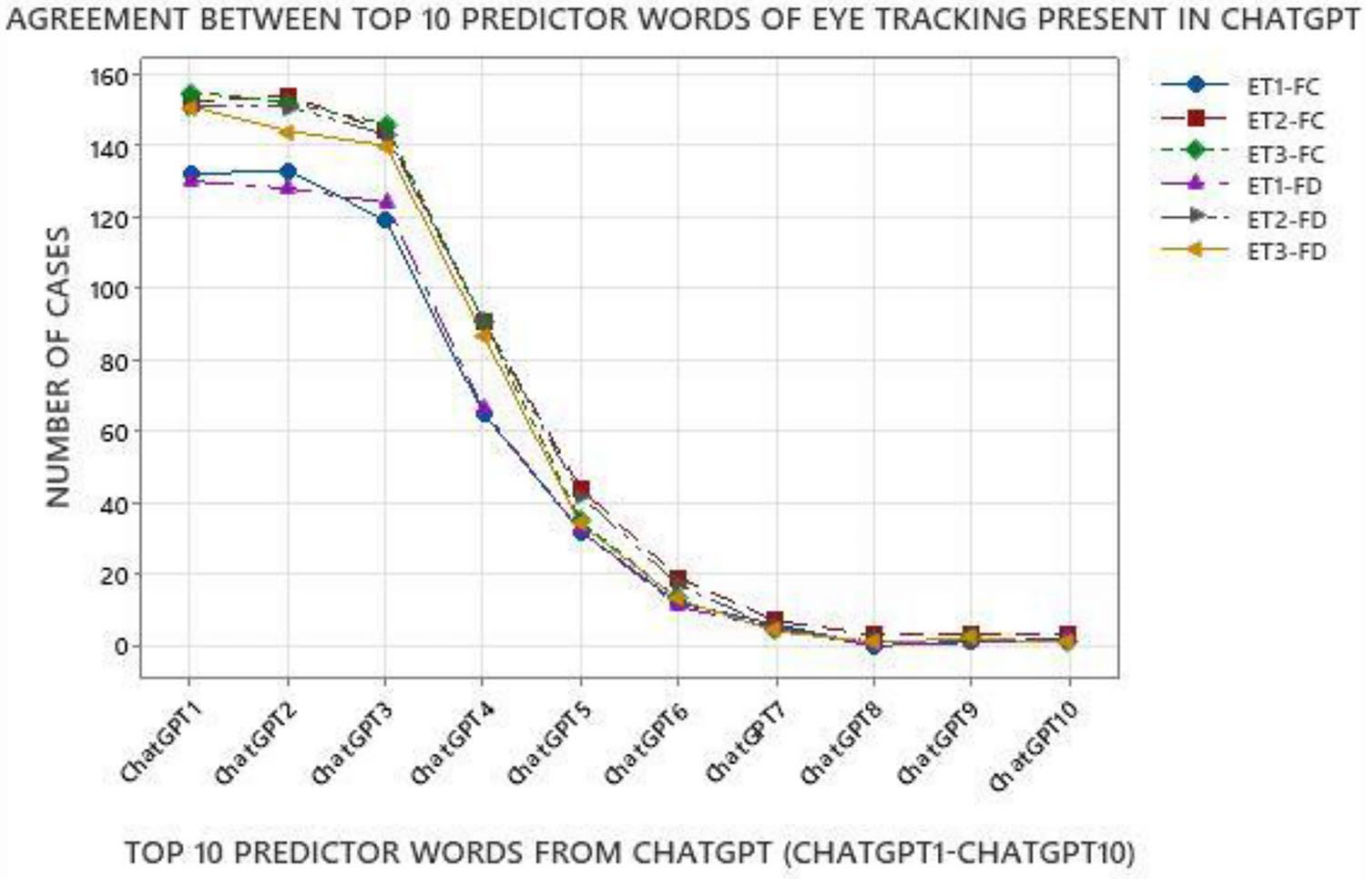
Agreement of top 10 predictor words of eye tracking (ET) present in ChatGPT.

Next, we compared the overlap between top predictor words used by ChatGPT and humans as shown in Fig. 5. The axes in Fig. 5 are organized similar to Fig. 4, with the Y-axis representing the number of cases from the 206-case dataset where the top words of humans and ChatGPT agreed, and the X-axis representing the top-10 ChatGPT words (ChatGPT1- ChatGPT10).

As shown in Fig. 5, the agreement between top words of ChatGPT and humans followed a similar overall trend as agreement between ML and humans shown in Fig. 4, with higher level of agreement with the first two/three words followed by a steady decline for later words. However, one of the noticeable differences between Figs. 4 and 5 is that there was a relatively steeper decline in agreement of top words of ChatGPT and humans as compared to ML and humans. Between humans and ML, the number of overlapping cases were in the range of 80–105 for ML4 and 80–110 for ML5, as shown in Fig. 4. For agreement between ChatGPT and humans, the number of overlapping cases were in the range of 60–90 for ChatGPT4 and 35–45 for ChatGPT5, as shown in Fig. 5. This indicates that while the top-3 predictor words aligned better with human reasoning, the later words had a relatively lower agreement with humans.

**Figure 6:**
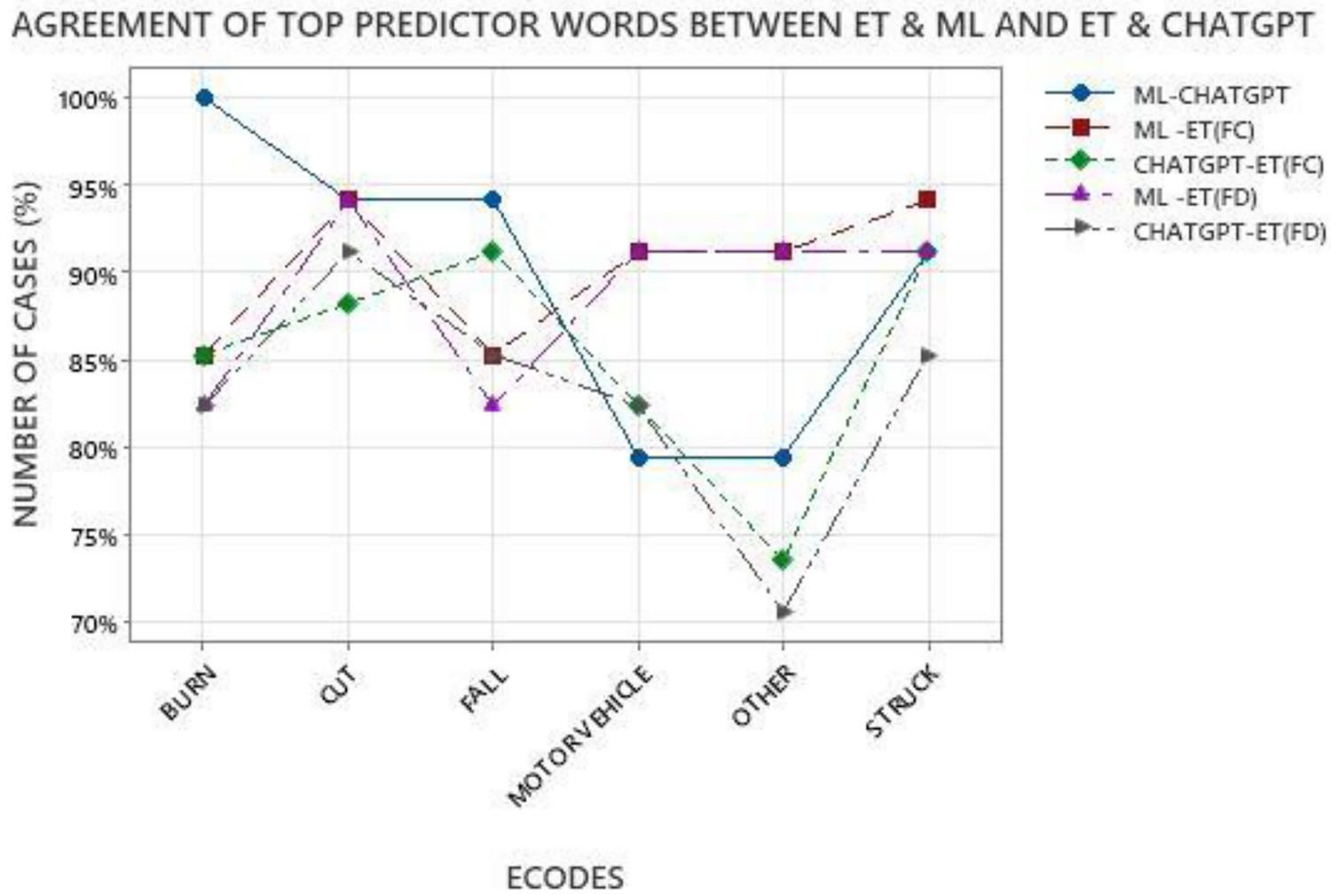
Agreement of top predictor words between eye tracking (ET) & machine learning (ML) and eye tracking (ET) & ChatGPT.

Next, we studied how the agreement of top predictor words between different approaches varied between different cause-of-injury codes as shown in Fig. 6. The intuition behind it was that the level of uniqueness of words used in the narratives associated with different cause-of-injury varied considerably, therefore, the top predictor words used by different approaches may vary based on the codes. As mentioned earlier, codes BURN and MOTORVEHICLE were relatively unique in nature, codes FALL, STRUCK and CUT were moderately unique, and the code OTHER was not very unique in nature. To examine the overlap of top predictor words between different approaches, we compared the following combinations: a) ML and ChatGPT (top-5 of ML and top-10 of ChatGPT) b) ML and Eye Tracking based on Fixation Count (ET(FC)), c) ChatGPT and ET(FC), c) ML and Eye Tracking based on Fixation Duration (ET(FD)), and d) ChatGPT and ET(FD). In Fig. 6, the X-axis shows all the six cause-of-injury codes, and the Y-axis represents the percentage of total number of cases for each category (34 cases of each category in the 206-case dataset) where the top-5 predictor words agreed between different text classification approaches. For this analysis, the data from the three sets of humans (ET1, ET2, and ET3) were combined together, and are represented as ET in Fig. 6.

Examining the overlap of top predictor words of ML and ChatGPT, we can see in Fig. 6 that the highest level of agreement was observed for code BURN followed by CUT, FALL, STRUCK, MOTORVEHICLE, and OTHER. As shown in Fig. 2, the prediction performances (Recall) of ML and ChatGPT were relatively close for codes CUT, MOTORVEHICLE, and FALL, and there was some difference in Recall for codes OTHER, STRUCK and BURN. Two interesting trends to note for the relatively unique codes BURN and MOTORVEHICLE were: a) while the Recall values for ML and ChatGPT were relatively different for BURN, the overlap between top words was high, and b) while the Recall values of ML and ChatGPT were close for MOTORVEHICLE, the overlap of top predictor words was low. One of the possible reasons might be the difference in approaches of interpreting the narrative words. The ML model was trained on thousands of injury narratives, so it was referring to the weight of words derived from the training set. The ChatGPT LLM model was not specifically trained on injury narratives, so the way in which it derived the relative importance of words was based on a more general vocabulary of words.

Between ML and humans, the highest overlap among top predictor words was observed for code CUT followed by STRUCK, MOTORVEHICLE, OTHER, FALL, and BURN, as shown in Fig. 6. Comparing the Recall values of ML and humans (ET1, ET2, and ET3), a relatively wider gap was observed for codes CUT, OTHER, and STRUCK. It is interesting to note that while there was a considerable difference in Recall values of ML and humans for codes CUT and STRUCK, the overlap between top predictor words was relatively high. A possible reason for it may be that since both these codes were moderately unique in nature, the same words may be used by humans or ML models to make different conclusions, i.e. selecting a different code.

Between ChatGPT and humans (ET(FC) and ET(FD)), the highest overlap between the top predictor words was observed for code FALL followed by codes CUT, BURN, STRUCK, MOTORVEHICLE, and OTHER. It can be observed from Fig. 6 that there was a considerable difference in the level of overlap between ChatGPT and humans based on fixation count ChatGPT-ET(FC) and fixation duration ChatGPT-ET(FD) for codes STRUCK and FALL as compared to other codes. No consistent trend was observed for variation of Recall values of ChatGPT and humans (ET1, ET2, and ET3) for different cause-of-injury codes.

## Discussion

In this study, we compared the performance and reasonings of a text classification task between non-expert humans, an advanced LLM, and a classical ML algorithm. The nature of text used in the study was not very generic (e.g., newspaper articles or customer reviews) but somewhat domain specific, containing injury narratives that had to be classified into six cause-of-injury categories. These injury narratives were noisy in nature making the task somewhat challenging. While the traditional ML model was trained on a large set of labelled injury narratives, the other approaches were not trained on injury classification. ChatGPT had a broader understanding of the English language through its diverse training sets and humans possessed language comprehension capabilities.

Results indicated that the ML model performed relatively better overall as compared to zero-shot LLM and non-expert humans. All approaches showed two consistent trends. First, the classification performance was better for cause-of-injury codes that were fairly unique in nature (BURN and MOTORVEHICLE), followed by moderately unique codes (FALL, STRUCK, and CUT), and codes that did not have a clear-cut definition (OTHER). Second, all the approaches performed relatively better for cases with low complexity, followed by medium and high complexity. It was also interesting to note that the ML model performed considerably better than ChatGPT and humans for cases with high complexity and those belonging to not so unique cause-of-injury codes (OTHER and STRUCK). This indicates that for a domain-specific text classification task, a traditional ML model trained on a large-size dataset can outperform non-expert humans and zero-shot LLM which are not trained on a similar dataset.

We compared the explainability of classification choices made by different approaches by comparing the words in the narrative that they focused on to make the classification decision. The results indicated that the top-3 words of ML had a higher degree of agreement with the top words used by humans and ChatGPT as compared to the top-4th and top-5th word. Similarly, for ChatGPT, the top-3 words overlapped to a greater extent with the words that humans focused on while comprehending and classifying the text, as compared to the later (n > 3) words. This suggests that the same top 1–3 words were used (typically the injury or object involved) for making the decisions by different approaches, although the conclusions may have been different for different approaches, particularly when the cause-of-injury did not have a unique definition, or the narrative was relatively complex.

As an additional baseline performance comparison of LLMs, we also studied the predictions from two other LLMs -Llama2 (21 billion parameters) and GPT-4. We compared the predictions and top-3 predictive words of Llama-2 and GPT-4 models with our base LLM model ChatGPT-3.5. The overall Recall of Llama-2 was 80.4% and GPT-4 was 68.6%, as compared to 76.5% for ChatGPT-3.5. We also noted there was a relatively high level of agreement between top words of Llama2 and ChatGPT-3.5. We examined the percentage of cases in the dataset where the top-3 words of Llama2 overlapped with the top-3 words of ChatGPT-3.5. Results indicated that for 75% cases in the dataset, the 1st word of Llama2 was present among the top-3 words of GPT-3.5, the overlap for Llama2’s 2nd word was 68% cases, and for the 3rd word it was 55% cases. Similarly, we examined the extent of overlap between top-3 words of ChatGPT4 and ChatGPT3.5 and observed the overlap to be 73% cases in the dataset for ChatGPT4’s 1st word, 60% cases for 2nd word, and 55% cases for the 3rd word. These results indicated that there was considerable overlap between the top words of both Llama2 and ChatGPT4 with ChatGPT-3.5, with the extent of overlap between Llama-2 and ChatGPT-3.5 being somewhat higher than that between ChatGPT-4 and ChatGPT-3.5.

Overall, our findings suggest that a simple and scalable machine learning model trained on large similar data can perform at par or better than untrained large language models and non-expert humans for a domain-specific text classification task. This study also had some limitations including: (a) the size of dataset and the number of participants were limited due to time and resource constraints, (b) limited number of models were compared in depth (LR for ML and ChatGPT for LLM) to examine the classification performance and reasoning, (c) the dataset used was domain-specific and the findings may not generalize for different types of textual datasets. To address these limitations, future studies can perform larger-scale eye-tracking user experiments with diverse datasets and different ML and LLM models. Future studies can also utilize the narrative word-importance measure captured during manual text annotation to adjust the feature weights of ML or LLM models for classification and interpretation purposes.

## Methods

### Human text classification

**Figure 7:**
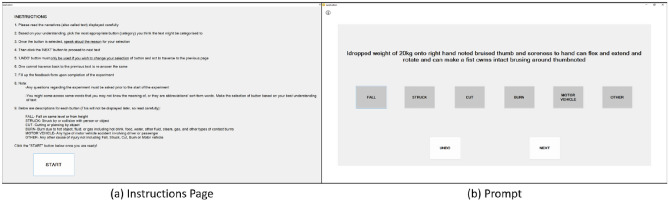
Injury Classification Software^35^.

An eye tracking study was conducted with 51 user participants consisting of 31 males and 20 females aged between 23 to 30 years, with a mix of 15 native and 36 non-native English speakers. The study was to read the injury related narratives and select the most appropriate cause-of-injury code using the injury classification software (Fig. 7). We used the Tobii Pro Fusion Eye Tracker with 250 Hz sampling frequency to capture gaze data and Tobii Pro Lab software to record ocular parameters for each participant. The Tobii Pro Lab uses the Velocity-Threshold Identification (IV-T) Fixation Filter algorithm^33^ to calculate angular velocity for each data point and depending on threshold values, the data points are classified as a fixation or a saccade^34^. Each participants’ ocular parameters such as fixation count (FC) and fixation duration (FD) for each word in the accident narrative, and selected cause-of-injury code for each narrative were recorded while they performed the text classification task. The recoded parameters were used to understand which words they focused on while reading and comprehending the narrative and making the selection of most applicable cause-of-injury code. The study was approved by the Institutional Review Board (IRB) at Purdue University. Taking part in the study was voluntary, and participants were allowed to choose to quit the study at any point in time. If they wished to participate, a consent form was required to be read and signed by each participant before the start of the study. Informed consent was obtained from all the 51 participants who took part in the study, and all of them were adults. All methods, as mentioned next, were performed in accordance with the relevant guidelines and regulations approved by the IRB.

The study was carried out in a well-lit indoor room. Each participant was called individually to the room and asked to position themselves towards the screen in a way they could comfortably read the content on screen and access the mouse. The Tobii Pro Fusion Eye Tracker was placed at the lower edge of the monitor that displayed the injury classification software. Participants were initially briefed about the process of the study and a trial session was given for them to be familiarized with the injury classification software. Then, the eye tracker was calibrated using the Tobii 9-point calibration routine. After the eye tracker was successfully calibrated, the injury classification software was displayed on screen. Participants were asked to read the instructions carefully and select the ‘START’ button when ready. Participants read the accident narrative displayed on screen and selected one of the six cause-of-injury codes that they felt was most appropriate.

The injury classification software (Fig. 7) consisted of an instructions page including meanings to each injury event cause and 12 prompts (Fig. 7a). Each prompt displayed one accident narrative and six possible cause-of-injury codes namely, CUT, FALL, STRUCK, BURN, MOTORVEHICLE, and OTHER (Fig. 7b). Description of each cause-of-injury code is given in Table 1. The participant had to select one of most applicable cause-of-injury codes. The buttons ‘NEXT’ and ‘UNDO’ on each prompt were used to navigate to next prompt and clear selection of cause-of-injury code to reselect the appropriate code respectively. Participants read the prompt and used the mouse to select the desired cause-of-injury code. We created 3 batches of 17 unique prompt-sets, each consisting of 12 prompts that were used for 51 participants. The 12 prompts consisted of a set of 2 unique narratives belonging to each of the six cause-of-injury codes. In total, we recorded 204 unique selections of cause-of-injury codes for each batch that included 34 narratives for each of the 6 cause-of-injury codes. Only 4 participants used the ‘UNDO’ button to reselect the desired cause-of-injury codes.

### ML text classification

For traditional ML model analysis, we used a Logistic Regression (LR) model trained on large-scale historical data with 120,000 cases of injury narratives associated with the above-mentioned 6 cause-of-injury codes. The training data was derived from the QISU database with cases recorded between 2013 and 2017, and the cause-of-injury codes were assigned by professional QISU coders. The trained LR model was used to predict the cause-of-injury codes on the same dataset of 204 narratives used in the human text classification study. For explainability analysis of the predictions made by the LR model, we used LIME based ELI5^5^ to identify the top-5 words in the narrative that the LR model used for making the cause-of-injury code prediction.

### LLM text classification

For the LLM analysis, we used the ChatGPT-3.5 model and created directive prompts for it to perform text classification on the same set of 204 accident narratives used for human and ML analysis. The model was also directed to report which were the top-10 words used by ChatGPT-3.5 to arrive at the decision about the cause-of-injury code for each narrative. The predictions made by the ML model, humans, and ChatGPT-3.5 were then compared with the original cause-of-injury codes assigned to accident narratives by the QISU professional coders. For explainability analysis, the top words and phrases used by each of three approaches were compared and analyzed, as presented in the next section.

For ChatGPT-3.5 we created prompts for it to perform text classification on accident narratives and report which were the top-10 words/phrases used by ChatGPT-3.5 to arrive at the decision about the cause-of-injury code. When the prompt (see *Prompt 2* below) to identify the top words for text classification was given to ChatGPT-3.5, the top-3 words/phrases were generated. However, the generated output consisted of more than one word grouped together as a single top word. Therefore, we separated each of the words from the top-3 words/phrases into the top-10 individual words to arrive at the decision. The prompts used to perform text classification using ChatGPT-3.5 are mentioned below:

*Prompt 1: For the narrative “*<*accident narrative*>*”, select the most appropriate type of injury from this list CUT, FALL, STRUCK, BURN, MOTORVEHICLE, OTHER TYPE?*

*Prompt 2: Which three words in the narrative are most indicative of the type of injury?*

## Data Availability

The data can be made available by contacting the corresponding author with a reasonable request.
